# Alcoholism

**Published:** 1886-03

**Authors:** W. C. Fisher


DANIEL'S

### Medical ★ Journal,

PUBLISHED MONTHLY AT

AUSTIN, TEXAS.

Vol. I.] MARCH, 1886. [No. 9.

*"Scribimus indocti, doctique!"*

### ORIGINAL ARTICLES.

☞ CONTRIBUTED EXCLUSIVELY TO THIS JOURNAL. ☜

*The Articles in this Department are accepted, and published with the understanding that we are not responsible for, nor do we indorse the views and opinions of the writers, by so doing.*

### ALCOHOLISM.

[Read before the Galveston Medical Club, at its meeting, February 8, 1886, by W. C. Fisher, M. D., and requested to be published.]
For Daniel's Texas Medical Journal.

MR. PRESIDENT AND GENTLEMEN:

IN the selection of a subject for the paper which I am to present to-night for your consideration, I have been somewhat at a loss to know what would be of most interest to you, so have decided to make a few remarks on the pathology, symtomology and treatment of that most common and serious disease known as Alcoholism.

This may be defined as a series of morbid phenomena, both mental and physical, produced by the use of alcoholic liquors. There are two great classes of conditions produced by alcohol; first, that which is known as acute alcoholism, which is the result of a large quantity taken in a short time; and second, chronic alcoholism, which is the result of the protracted use of large quantities of this subtle agent.

We will first deal with the acute form. It is useless for us to discuss that condition, generally known as drunk, which will, on the withdrawal of the liquor,and a good sleep, correct itself, in the course of twenty-four or forty-eight hours. without any medical treatment. That condition known as "dead drunk," often resembling either opium narcosis or apoplexy, we will mention, to show the diagnostic difference between them. In this state we often have the most profound sleep, relaxation of the muscles, congested face, blue lips, contracted pupils, slow stertorous breathing, slow bounding pulse ; and the breath, as well as the whole body, gives forth an aroma very suggestive of the alcoholic beverage which had been taken. When a case is brought to us. it is very necessary to make all inquiries as to the individual's habits, customs, etc. ; in other words, obtain all the information we can before giving an opinion, or prognosticating the result. We often have to deal with a hybrid condition, where the individual who has been freely indulging, seeks relief from his condition by the opium route ; then it is, of course, impossible to tell which is producing the coma, and only time and patience will tell the sequel. In opium narcosis there is no aroma given off from the breath, unless the patient has taken either laudanum or paragoric, and these are so familiar to every physician's organ of smell that he would not doubt for a moment the poison taken ; and then the narcosis produced by opium or any of its preparations is not so prolonged as that from alcohol, and unless relieved by the proper antidotes and therapeutical measures in a very short while, will take the unfortunate individual to the happy hunting ground. Apoplexy is generally very sudden in its invasion, and is accompanied most often by dilation of the pupils ; but, of course, there are exceptions to this rule, depending on the portion of the brain affected ; there is also generally paralysis, which is absent in the two other conditions.

The treatment of this condition is often best carried out by letting the patient sleep it off; but where the coma is very profound, it may be necessary to use such derivatives as a drop or two of croton oil on the tongue, a fly blister to the nucha, and a capsicum enema; if the respiration be much embarrassed, it may be necessary to stimulate the phrenic nerves with an electric current; this, however, will seldom be the case. Then the chances are that in a reasonable time the individual will awake from his Rip Van Winkle sleep, and call for his toddy; in other words, the prognosis is good.

Another condition under the head of acute alcoholism, is that known as acute alcoholic delirium, or mania-a-potu, which we observe in a highly nervous individual after heavy debauch, and especially in one who has had serious trouble, such as great financial losses, family troubles, and in one who has been greatly enervated by loss of sleep, deficient alimentation or overwork. In such a person we often have the wildest delirium, accompanied by hallucinations and illusions; and unless the patient be controlled, he may do injury to himself or those around him. This is to be distinguished from what is known as delirium tremens in that it is not accompanied by the characteristic tremor, and the pathological changes which exist in the latter, but is due to the direct action of the alcohol on the brain, which does not suffer any appreciable lesion. Unless this condition soon be controlled, it may possibly end in alcoholic mania, especially in one with a predisposition. The indication for treatment is to quiet the patient as soon as possible by producing sleep, and this is best done by administering hyoscimia in 1-10 or 1-40 gr. doses, hypodermatically, full doses of the bromides and chloral; of the latter drug I am very cautious, for at times it has a very depressing action on the heart, and, besides, is very uncertain; it is always well to combine it with full doses of digitalis and capsicum, not only as a precautionary measure, but also because they have a sedative action in alcoholism.

We will now deal with that second great condition, known as chronic alcoholism, the definition of which I gave in my introduction. In the victim of this disease we have before us a number of pathological changes; scarcely an organ or tissue escapes, and the symptoms are numerous and characteristic. I will not tax your patience with the pathological phenomena which are observed in the dead house, for they are too well known to all of us who have held many autopsies, and are thoroughly set forth in all standard words on pathology; and I will endeavor to be brief with the symptoms that we most often observe, for I

already feel that I have tried your patience beyond endurance. The subject of this unfortunate disease soon loses respect for himself and all the world, and his only thought is for something to drink; after a while his memory grows weaker, his judgment becomes less accurate, and he is unable to concentrate his thoughts, to fix his attention, associate ideas—in other words, he is failing mentally. He is neglectful of both his family and business affair, and becomes careless regarding his personal appearance. He daily feels the need of more stimulant, in order to "*keep out.*" Soon his appetite fails him, and gastritis is set up, which is attended by retching and vomiting of glairy mucus, ete.; this is especially the case on getting up in the morning, and is somewhat relieved after the morning toddy. The tongue is now apt to be red, and is very tremulous; in fact, there is no general tremor. The face, as a rule, becomes bloated, and heavy looking, with the characteristic nasal appendage; the lower extremities are œdematous, owing to changes in the liver; and the abdomen for the same reason may become ascitic. The patient experiences peculiar sensations, such as drumming in the head, ringing in the ears, and, later, we have such changes in the nervous system that the patient suffers with paresis and with hallucinations, especially on first retiring on rising. The heart is apt to become fatty, and the kidneys, like the liver, to become cirrhotic.

The treatment in the early stages of the disease, before organic changes have taken place, may be most beneficial, and curative, provided we can get the co-operation of the patient, and his consent to give up liquor; such is very seldom our good luck. We have many promises made and broken, and we, as doctors, have very little confidence in them. However, occasionally a drunkard will stop in time, and, by the judicious management of the physician, he may be made an ornament to society and a useful citizen. If possible, remove him from the enticing influences of his numerous friends, who worship at the shrine of Bacchus, and make his surroundings as pleasant as possible; and if his "craving" for liquor be great, give him an occasional toddy, or glass of wine; a mild tonic, and a good stimulating diet.

When organic changes, such as cirrhosis of the liver and kidneys, brain lesions and heart affections occur, their only treatment is by the gradual withdrawal of the prime etiological factor, in the hope of staying the disease, and direct our treatment to the palliation of the symptoms as they arise, and the judicious alimentation of the patient. A cure in these cases is impossible, but we may, by careful treatment, make their sufferings comparatively slight and their life much more enjoyable.

We will now consider briefly that state called Delirium Tremens, which, in the language of one of our most eminent pathologists, is "an acute alcoholic delirium due to an unusual consumption of spirits in a subject of chronic alcoholism; or occasionally it is produced by the sudden withdrawal of the accustomed amount of stimulus; the stomach is disturbed, food and drink are rejected, and hence the nervous system is left unsupplied; an attack may also be induced by some strong moral emotion or excitement, or by an accident or injury."

After a continued debauch or much mental or physical excitement, the trembling of the chronic condition is increased; the individual is much excited, has a wild expression, no appetite, is unable to sleep, or only gets "cat naps," and wakes with a wild start; he has hallucinations and illusions of the most peculiar kind, and of every conceivable variety; sometimes they are of a most agreeable nature, at others they are most horrible, and throw the patient into the wildest state of excitement, and we are told that there are, besides visual illusions, those of smell and hearing. At first the pulse is rapid, but rather full, but in the course of several days (perhaps two or three) the pulse becomes weak, and shows marked depression; and now the patient has a considerable rise of temperatnre, with a dry, cracked tongue, low muttering delirium, and involuntary discharges. At this time pneumonia is apt to complicate the case, and is frequently the immediate cause of death; rupture of some cerebral artery is apt to occur, and also cause a fatal termination. The duration, provided no complications arise, is generally from ten days to two weeks, when the patient either recovers or dies. In some instances it is prolonged considerably.

The prognosis, provided the patient has not been too long addicted to alcoholism, and is well nourished, is good, if he is judiciously treated. There are many different plans of treatment suggested by different authors—some believing in the absolute withdrawal of stimulants, and the use of cerebral sedatives; others in the moderate allowance of alcohol; others in heroic doses of digitalis, and a few give immense quantities of capsicum, and pay strict attention to the patient's alimentation.

A plan that I have found most successful is to put my patient in a room by himself, attended by *one good, reliable* nurse. If there is much irritability of the stomach, I try every means in the world to quiet it as soon as possible, so that I can give sufficient nourishment; if the stomach rejects everything, I resort to rectal alimentation. In some cases I find it advisable to allow some little liquor, in order to quiet the stomach, and enable the patient to digest that which he eats, and also for the purpose of diminishing his "craving;" and when there is much depression, as indicated by the heart's action, it becomes absolutely necessary.

In the majority of cases which I have seen, together with our esteemed President, in hospital practice, we found the total withdrawal of alcohol, firm nurses, and dram doses of tr. capsicum and valerian work wonders, together with good nourishment. The capsicum tones up the stomach, and also has a sedative action on the brain, and is itself a most excellent stimulant.

Now, gentlemen, thanking you for your attention and kind forbearance, I will bring this uninteresting paper to a close.

### ON THE TREATMENT OF ORCHITIS BY DIVISION OF THE TUNICS OF THE TESTICLE.

*By A. S. Wolf, M.D., Brownsville, Texas.*

For Daniel's Texas Medical Journal.

